# Meta-analysis study to evaluate the association of MTHFR C677T polymorphism with risk of ischemic stroke

**DOI:** 10.6026/97320630013214

**Published:** 2017-06-30

**Authors:** P.A. Abhinand, M. Manikandan, R. Mahalakshmi, P.K. Ragunath

**Affiliations:** 1 Department of Bioinformatics, Sri Ramachandra University, Porur, Chennai-600 116, India

**Keywords:** MTHFR, MTHFR C677T, ischemic stroke, meta-analysis, folic acid

## Abstract

Ischemic stroke is a condition characterized by reduced blood supply to part of the brain, initiating the ischemic cascade, leading to dysfunction of the brain tissue in that area. It is one of the leading causes of death and disability and is estimated to cause around 5.7 million deaths worldwide. Methyl tetra hydro-folate reductase (MTHFR) is a rate limiting enzyme in the methyl cycle which catalyzes the only biochemical reaction which produces 5, Methyl tetra hydro folate, the co-substrate for the re-methylation of homocystiene to produce methionine. MTFHR C677T is a common mutation of MTHFR and those homozygous for the MTFHR C677T produce a thermo-labile form of the protein with drastically reduced catalytic activity resulting in elevated plasma homocystiene levels - a common risk factor for cardiovascular diseases. However, the role of MTHFR C677T in ischemic stroke remains unclear. To evaluate this association, we carried out a meta-analysis of existing published studies, which included 72 studies involving 12390 cases and 16274 controls. The forest plot was made to evaluate the overall risk of the mutation in the etiology of Ischemic Stroke. The overall Odds- ratio of the study was found to be 1.319 for random effects model, revealing a ∼32% increased risk of Ischemic stroke in the presence of MTHFR C667T mutation compared to controls. Publication bias in the study was analyzed using funnel plot which revealed that only 7 studies out of the 72 contributed to publication bias. These 7 studies were excluded and Meta-analysis was repeated for 65 studies and overall odds-ratio was 1.306, which showed that there was a 30% higher risk of Ischemic stroke in the presence of MTHFR C667T.

## Background

Stroke is a clinical condition characterized by poor blood flow to the brain resulting in cell death. It is of 2 major types: ischemic, due to lack of blood flow, and hemorrhagic, due to bleeding. Ischemic stroke is a clinical condition characterized by reduced blood supply to part of the brain, initiating the ischemic cascade, leading to dysfunction of the brain tissue in that area. The reduced blood flow can be caused by Thrombosis, Embolism, Systemic hypo perfusion or venous thrombosis. [1] Stroke is one of the leading causes of death and disability in India and world over. In 2005, ischemic stroke is estimated to cause around 5.7 million deaths worldwide and 87% of these deaths were in low income and middle-income countries. [2] The estimate adjusted prevalence rate of stroke range, 84-262/100,000 in rural and 334- 424/100,000 in urban areas. The incidence rate is 119-145/100,000 based on the recent population based studies. [3]

Methyl tetra hydro folate reductase (MTHFR) gene which is located on chromosome 1 (1p36.3) encodes a 77 kDa dimeric protein of the same name, which is a rate-limiting enzyme in the methyl cycle. [4] It catalyzes the only biochemical reaction, which produces 5, Methyltetrahydrofolate, the co-substrate for the remethylation of homocystiene to produce methionine. [5] MTFHR C677T (C → T substitution at bp 677) is a common mutation of MTHFR causing an Alanine to Valine substitution at the 222nd position in the encoded protein product. People who are homozygous for the MTFHR C677T mutation produce a thermolabile form of the protein with drastically reduced catalytic activity resulting in elevated homocystiene levels in the plasma. [6, 7] Even a modest increase in plasma homocysteine has been known to be risk factor for cardiovascular diseases. [8, 9] However, its role in stroke remains unclear. Although most case-control studies suggest a positive association between elevated plasma homocysteine and stroke, nested case-control studies to establish such an association are rare and are limited by the availability of previous studies. [10]

Meta-analysis is a powerful statistical technique involving analysis of a large collection of analysis results from individual studies for the purpose of integrating the findings. [11, 12] It is a quantitative and formal epidemiological study design used to systematically assess the results from previous research to derive conclusions about that body of research. [13] We performed an updated systematic review and cumulative meta-analysis of available data and quantify the stroke risk associated with the 677T allele with a sufficient sample size to address these power limitations.

## Methodology

### Systematic Literature Survey

Electronic databases (PUBMED, Cochrane library and Google Scholar) were searched until March 2012 for all case - control studies evaluating MTHFR C677T gene polymorphism and ischemic stroke in humans.

In PUBMED the following queries were used for enlisting all the eligible studies

((׳M׳ OR ׳m׳) AND C677T AND ׳I׳ AND (׳H׳ OR ׳h׳) ((׳M׳ OR ׳m׳) AND C677T AND ׳CI׳ AND (׳H׳ OR ׳h׳) ((׳M׳ OR ׳m׳) AND C677T AND ׳BI׳ AND (׳H׳ OR ׳h׳)

M = Methylenetetrahydrofolatereductase; m = MTHFR; I=Ischemic Stroke; CI = Cranial infraction; BI = Brain Ischemia H = Homo sapiens; h = human

All published manuscripts including letters, previous metaanalyses, and abstracts were searched. The retrieved studies were examined thoroughly to assess their appropriateness for inclusion. The search results were limited to human. All languages were searched initially, but only articles in English language were selected. The references of all computer-identified publications were searched for any additional studies, and the MEDLINE option-related articles were used for all the relevant articles.

### Inclusion and Exclusion Criteria

Studies were included if: (a) study design was case - control and (b) had confirmed diagnosis of ischemic stroke with magnetic resonance imaging (MRI) or computed tomography (CT). A standardized data collection form was used for data extraction; this form mainly included the following content: (i) Name of the first author, year of publication, country, and racial descent; (ii) Demographics, number of cases and controls, and source of cases and controls; (iii) Distribution of genotypes and alleles; (iv) Hardy-Weinberg equilibrium

### Meta-Analysis

**Figure 1:**
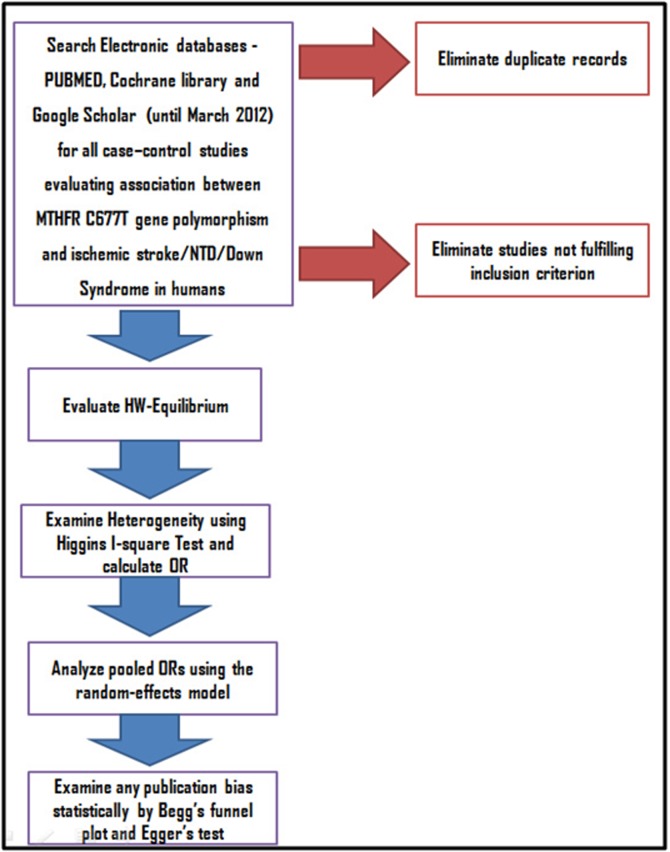
Steps Involved in Meta-analysis to study the association of MTHFR C677T polymorphism with risk of Ischemic Stroke

The Comprehensive Meta-Analysis Version 3.0 performed all statistical analyses. Two-sided p values less than 0.05 were considered statistically significant. For the control groups for each study, the observed genotype frequencies of the MTHFR C677T polymorphism were evaluated for Hardy-Weinberg equilibrium. The strength of the association between the MTHFR C677T polymorphism and Ischemic Stroke was assessed by the odds ratios (ORs) with 95% CIs. The pooled ORs of Patients with Ischemic Stroke vs. Healthy controls were calculated for the dominant model (CT + TT vs. CC). The evaluation of the metaanalysis results included a test for heterogeneity, an analysis of the sensitivity, and an examination for publication bias. Considering possible heterogeneity between studies, I2 metric were conducted, p < 0.10 and I2> 50% were considered to indicate the existence of significant heterogeneity. [14] If the heterogeneity test result returned p > 0.1, the pooled ORs were analyzed using the random-effects model [15], or else, the fixed effects model was used. [16] Sensitivity analyses were also performed after sequential removal of each study. Lastly, Begg׳s funnel plot and Egger׳s test were used to examine statistically any publication bias [17, 18]. The overall methodology is depicted in Figure 1

## Results and Discussion

**Figure 2:**
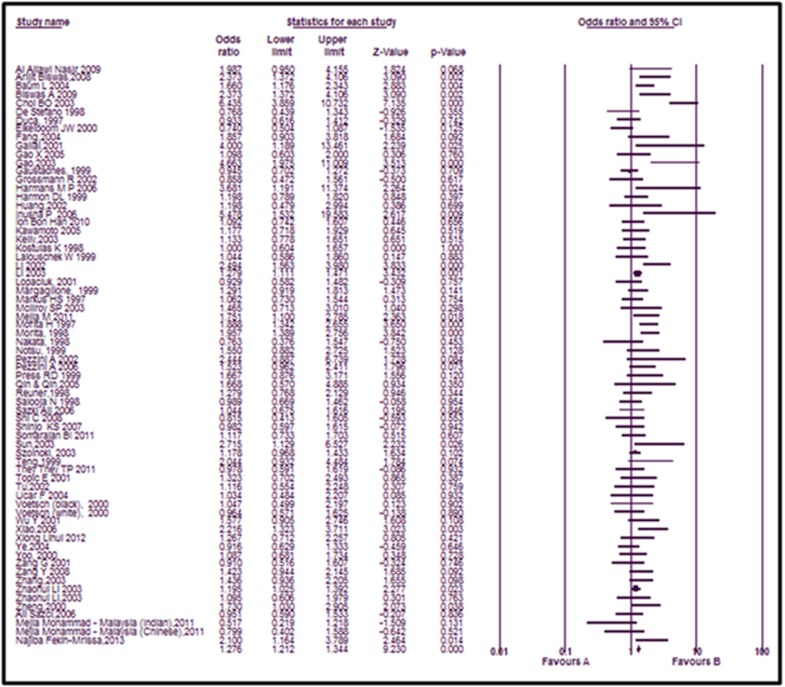
Forest plot and pooled ORs of risk from studies investigating MTHFR C677T polymorphism and ischemic stroke for Dominant model

The current study investigated the association between risk of ischemic stroke and MTHFR C677T polymorphism. Study revealed that the presence of MTHFR C677T significantly increases the risk of ischemic stroke. Numerous studies have been carried out across the globe to determine the association between MTHFR C677T polymorphism and ischemic stroke; however, the association remains inconclusive. With an aim to accurately quantify this association, we carried out a metaanalysis of existing published studies, which included 72 studies involving 12390 cases and 16274 controls (Shown in Table 1). The forest plot was made to evaluate the overall risk of the mutation in the etiology of Ischemic Stroke. The overall Odds- ratio of the study was found to be 1.276 for fixed effect model and 1.319 for random effects model, which showed that there was a ~32% increased risk of Ischemic stroke in the presence of MTHFR C667T mutation compared to controls. The Forest plot is depicted in Figure 2 The findings from the current meta-analysis study are in agreement with both the previously carried out meta-analyses on evaluating association between risk of ischemic stroke and MTHFR C677T polymorphism. The study concurs with results from Li et al. (2004) [19] which included 19 case - control studies involving 2223 cases and 2936 controls where this polymorphism was found to be potentially associated with the risk of ischemic stroke. [19] Our results also agree with the findings of Kumar et al. (2015) [20] comprising of 6310 patients and 8297 controls. [20] Publication bias in the study was analyzed using funnel plot, which revealed that only 7 studies out of the 72 contributed to publication bias. These 7 studies were excluded and Metaanalysis was repeated for 65 studies. The overall odds-ratio was found to be 1.306 that showed that there was a 30% higher risk of Ischemic stroke in the presence of MTHFR C667T mutation compared to controls.

## Conclusion

This current study is the largest meta-analysis consisting of 72 studies, carried out to evaluate the association between MTHFR C677T polymorphism and the risk of ischemic stroke. The study done showed that the polymorphism significantly increased (∼30%) the risk of ischemic stroke. The study further suggests the importance of MTHFR genotyping for identifying patients susceptible for risk of ischemic stroke and for preventing and managing stroke cases. The study findings have a clear implication on health policy makers to enable increased intake of Levomefolic acid to reduce the risk of ischemic stroke. Bigger prospective studies with correction for multiple comparisons are essential for further validating the study findings.

## Figures and Tables

**Table 1:** Characteristics of studies included in the meta-analysis with respective sample sizes

Study Name	Year of Study	Origin	Wild Type Case (CC)	Mutant Case (CT+TT)	Wild Type Control (CC)	Mutant Control (CT+TT)	Hardy Weinberg Equilibrium
Al AllawiNasir	2009	Iraq	26	44	27	23	Yes
Alluri RV	2005	India	50	25	48	1	Yes
ArijitBiswas	2008	India	67	53	90	30	Yes
Baum L	2004	China	125	116	195	109	Yes
Biswas A	2009	India	67	53	90	30	Yes
Choi BO	2003	Korea	62	133	90	30	Yes
D. Arsene	2011	Romania	37	30	17	43	Yes
De Stefano	1998	Italy	28	44	65	133	Yes
Duca	1997	Italy	62	118	74	151	Yes
Elkelboom JW	2000	Australia	106	113	84	121	Yes
Fang,	2004	China	15	39	40	56	Yes
Gallai	2001	Italy	5	20	15	15	Yes
Gao X	2005	China	30	70	32	68	Yes
Gao,	2003	China	13	67	19	21	Yes
Gaustadnes,	1999	Denmark	107	100	545	539	Yes
Grossmann R	2002	Germany	73	20	141	45	Yes
Harmans M P	2006	Belgium	4	19	62	80	Yes
Harmon DL	1999	Ireland	74	100	86	97	Yes
Huang	2002	China	11	28	16	34	Yes
Inusha P	2006	India	23	9	56	4	Yes
Ion Bon Han	2010	Korea	75	188	71	163	Yes
Kawamoto	2005	Japan	33	64	91	150	Yes
Kelly	2003	North America	102	141	91	111	Yes
Kostulas K	1998	Sweden	76	50	76	50	Yes
Lalouschek W	1999	Austria	38	58	39	57	Yes
Li	2002	China	58	85	97	57	Yes
Li	2003	China	520	1303	619	1213	Yes
Lopaciuk	2001	Poland	51	49	117	121	Yes
Margaglione	1999	Italy	53	149	326	710	Yes
Markus HS	1997	UK	162	183	78	83	Yes
Mcllroy SP	2003	Ireland	39	24	50	21	Yes
Mejia M	2011	Malaysia	69	81	85	57	Yes
Mejia Mohammad	2011	Malaysia (Indian)	25	17	19	25	Yes
Mejia Mohammad	2011	Malaysia (Chinese)	33	39	25	37	Yes
Mejia Mohammad	2011	Malaysia (Malay)	23	13	13	23	Yes
NajibaFekih-Mrissa	2013	Tunisia	35	49	60	40	Yes
Pezzini A	2002	Italy	9	22	18	18	Yes
Pezzini A	2005	Italy	46	117	60	98	Yes
Pezzini A	2006	Italy	51	123	60	95	Yes
Press RD	1999	USA	56	80	28	24	Yes
Qin & Qin	2005	China	11	39	8	17	Yes
Reuner	1998	Germany	37	54	85	97	Yes
Salooja N	1998	UK	114	128	81	92	Yes
Sazki Ali	2006	Turkey	52	68	115	144	Yes
Shi C	2006	China	23	74	20	79	Yes
Shinjo KS	2007	Brazil	55	72	54	72	Yes
Somarajan BI	2011	India	137	70	129	59	Yes
Sun	2003	China	8	53	25	61	Yes
Szolnoki	2003	Hungary	415	452	386	357	Yes
Teng	1999	China	12	50	26	53	Yes
They They TP	2011	Morocco	48	43	95	87	Yes
Topic E	2001	Croatia	25	31	64	60	Yes
Tu	2002	China	24	45	25	42	Yes
Ucar F	2004	Turkey	15	15	123	119	Yes
Voetsch	2000	Brazil (black)	22	17	61	45	Yes
Voetsch	2000	Brazil (white)	47	67	48	71	Yes
Wu Y	2001	Japan	23	54	92	137	Yes
Xiao,	2006	China	49	113	49	51	Yes
XiongLihui	2012	China	35	54	46	56	Yes
Ye,	2004	China	132	68	192	108	Yes
Yoo	2000	South Korea	41	81	77	140	Yes
Zang G	2001	China	40	62	37	63	Yes
Zang Y	2008	China	49	196	74	208	Yes
Zhang	2003	China	45	189	66	193	Yes
Zhaohui Li	2003	China	389	931	610	1222	Yes
Zhaohui Li	2003	China	29	71	34	76	Yes
Zheng	2000	China	43	72	62	60	Yes
Ali Sazci	2006	Turkey	42	50	115	144	Yes

**Figure 3:**
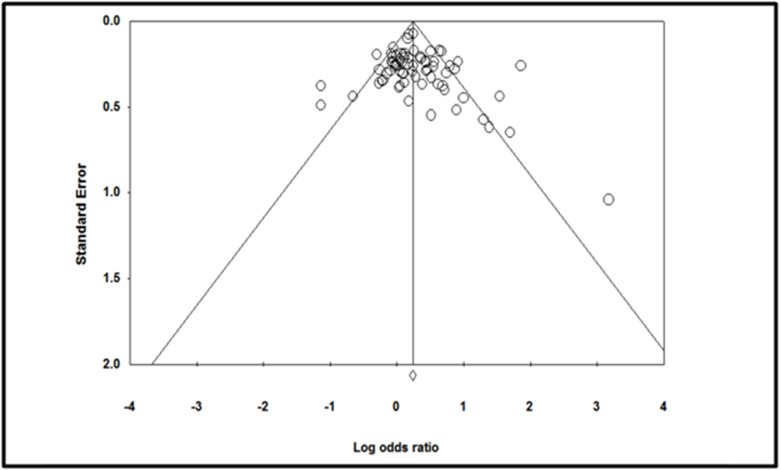
Funnel plot of Standard error by Log odds ratio for assessing publication bias
